# Axial length and its associations in the Ural Very Old Study

**DOI:** 10.1038/s41598-021-98039-z

**Published:** 2021-09-16

**Authors:** Mukharram M. Bikbov, Gyulli M. Kazakbaeva, Ellina M. Rakhimova, Iuliia A. Rusakova, Albina A. Fakhretdinova, Azaliia M. Tuliakova, Songhomitra Panda-Jonas, Timur R. Gilmanshin, Rinat M. Zainullin, Natalia I. Bolshakova, Kamilia R. Safiullina, Ainur V. Gizzatov, Ildar P. Ponomarev, Dilya F. Yakupova, Nail E. Baymukhametov, Nikolay A. Nikitin, Jost B. Jonas

**Affiliations:** 1 Ufa Eye Research Institute, 90 Pushkin Street, Ufa, 450077 Bashkortostan Russia; 2 Institute of Clinical and Scientific Ophthalmology and Acupuncture Jonas & Panda, Heidelberg, Germany; 3 Department of Ophthalmology, Medical Faculty Mannheim of the Ruprecht-Karls-University of Heidelberg, Theodor-Kutzerufer 1, 68167 Mannheim, Germany; 4 Institute of Molecular and Clinical Ophthalmology Basel, Basel, Switzerland

**Keywords:** Anatomy, Nervous system, Sensory systems, Visual system, Object vision, Diseases, Eye diseases, Refractive errors

## Abstract

To assess the distribution of axial length as surrogate for myopia and its determinants in an old population, we performed the Ural Very Old Study as a population-based cohort study. Out of 1882 eligible individuals aged 85 + years, the Ural Very Old Study performed in an urban and rural region in Bashkortostan/Russia included 1526 (81.1%) individuals undergoing ophthalmological and medical examinations with sonographic axial length measurement. Biometric data were available for 717 (47.0%) individuals with a mean age of 88.0 ± 2.6 years (range 85–98 years; 25%). Mean axial length was 23.1 ± 1.1 mm (range 19.37–28.89 mm). Prevalences of moderate myopia (axial length 24.5–< 26.5 mm) and high myopia (axial length ≥ 26.5 mm) were 47/717 (6.6%; 95% CI 4.7, 8.4) and 10/717 (1.4%; 95% CI 0.5, 2.3), respectively. In multivariable analysis, longer axial length was associated (coefficient of determination r^2^ 0.25) with taller body height (standardized regression coefficient beta:0.16;non-standardized regression coefficient B: 0.02; 95% confidence interval (CI) 0.01, 0.03; *P* < 0.001), higher level of education (beta: 0.12; B: 0.07; 95% CI 0.02, 0.11; *P* = 0.002), and lower corneal refractive power (beta: − 0.35; B: − 0.23; 95% CI − 0.28, − 0.18; *P* < 0.001). Higher prevalence of moderate myopia, however not of high myopia, was associated with higher educational level (OR 1.39; 95% CI 1.09, 1.68; *P* = 0.007) and lower corneal refractive power (OR 0.77; 95% CI 0.63, 0.94; *P* = 0.01). In this old study population, prevalence of moderate axial myopia (6.6% versus 9.7%) was lower than, and prevalence of high axial myopia (1.4% versus 1.4%) was similar as, in a corresponding study on a younger population from the same Russian region. Both myopia prevalence rates were higher than in rural Central India (1.5% and 0.4%, respectively). As in other, younger, populations, axial length and moderate myopia prevalence increased with higher educational level, while high myopia prevalence was independent of the educational level.

## Introduction

The ocular axial length is a principal biometric measure of the eye and the most important determinant of axial ametropias, i.e. myopia and hyperopia. It is associated with ocular disorders and diseases such as primary angle-closure glaucoma, diabetic retinopathy, age-related macular degeneration, strabismus and amblyopia in the case of axial hyperopia, and with rhegmatogenous retinal detachment, myopic macular degeneration and high myopia-associated optic neuropathy in the case of axial myopia^1–4^. In the recent decades, the prevalence of axial myopia has markedly increased and the prevalence of axial hyperopia has subsequently decreased^5^. It has thus been anticipated that the prevalences of ocular disorders associated with axial length will change in the near future. In particular high myopia as the main risk factor for pathologic myopia has been estimated to be, or to become, the most common cause for irreversible vision impairment and blindness^6, 7^. Although axial length as the main measure of axial myopia has already been assessed in various population-based studies in several continents, data about the distribution of axial length in the very old population have been missing so far^8–14^. Knowledge about the prevalence of axial myopia, in particular of high myopia, in an elderly, yet unexplored population could give insight into the associations of axial moderate and high myopia with other ocular and systemic parameters in such a study sample, and, by comparing the prevalence of high myopia between such an elderly population and a normal-aged population could potentially give hints for the future development of the prevalence of high myopia. We therefore conducted this survey to examine the distribution of axial length and its determinants in a study population aged 85 + years^15^. To avoid a referral bias, we recruited the participants in a population-based manner.

## Methods

The Ural Very Old Study (UVOS) is a population-based study performed in the rural region of the Karmaskalinsky District in a distance of 65 km from the capital Ufa, and in the Kirovskyi discrict—an urban region of the capital Ufa in the Republic of Bashkortostan/Russia. The study conducted between November 2017 and December 2020, was approved by the Ethics Committee of the Academic Council of the Ufa Eye Research Institute confirming that all methods were performed in accordance with the relevant guidelines and regulations, and informed written consent was obtained from all participants. Inclusion criteria were an age of 85 + years and living in the study regions. There were no exclusion criteria.

Out of 1882 eligible individuals, 1526 (81%) persons participated in the study. The eligible individuals, recruited in a census manner, included the inhabitants of three private small retirement homes in the urban study region (with about 45 study individuals from all three homes together). There were no retirement homes in the rural study region. The participation rate did not vary markedly between the urban group [1238 (81.3%) out of 1523 individuals] and the rural group [288 (80.2%) out of 359 individuals]. According to the census carried out in Russia in 2010, the composition of the UVOS population with respect to gender and age corresponded to the gender and age distribution in the Russian population beyond an age of 85 + years, with a marked preponderance of females^16^.

All study participants underwent a standardized interview conducted by trained social workers with about 300 questions on the socioeconomic background, diet, smoking, alcohol consumption, physical activity, quality of life and quality of vision, symptoms of chronic obstructive pulmonary disease, asthma, kidney disease and orthopedic disorders, history of any type of injuries and inter-personal violence, health assessment questions, and history of major medical disorders. The questions had been validated in previous investigations such as the Folstein test, Zung’s self-rated depression scale, and the National Eye Institute Visual Functioning Questionnaire-25 (VFQ-25) (Table 1)^17–19^.

**Table 1:** Significant associations (univariate analysis) between axial length and systemic parameters in the Ural Eye and Medical Study.

	*P*-value	Standardized regression coefficient beta	Non-standardized regression coefficient B	95% confidence interval of B
Age	− 0.03	− 0.01	− 0.04, 0.02	0.50
Gender (men/women)	0.16	0.39	0.21, 0.57	< 0.001
Region of habitation (rural/urban)	0.08	0.41	0.03, 0.78	0.04
Body height	0.23	0.03	0.02, 0.04	< 0.001
Body weight	0.08	0.01	0.00,0.01	0.04
Body mass index	− 0.08	− 0.02	− 0.04, − 0.001	0.04
Level of education	0.17	0.10	0.06, 0.14	< 0.001
History of asthma	0.09	0.66	0.12, 1.20	0.02
History of dementia	− 0.08	− 0.44	− 0.84, − 0.04	0.03
High-density lipoproteins (mmol/L)	− 0.09	− 0.14	− 0.25, − 0.03	0.01
Cholesterol (mmol/L)	− 0.09	− 0.08	− 0.14, − 0.01	0.02
Manual dynamometry, right hand	0.12	0.02	0.01, 0.03	0.002
Manual dynamometry, left hand	0.11	0.02	0.01, 0.03	0.004
Mini mental test	0.10	0.02	0.002, 0.04	0.03
**Ophthalmological parameters**				
Refractive error, spherical value (diopters)	− 0.19	− 0.08	− 0.11, − 0.05	< 0.001
Refractive error, cylindrical value (diopters)	− 0.12	− 0.07	− 0.11, − 0.02	0.004
Refractive error, spherical equivalent (diopters)	− 0.20	− 0.07	− 0.10, − 0.04	< 0.001
Corneal refractive power	− 0.35	− 0.24	− 0.29, − 0.19	< 0.001
Dry eye, definition #2	0.09	0.35	0.07, 0.62	0.02

The physical examinations consisted of measurements of anthropomorphic parameters such as body height and weight, arterial blood pressure and pulse rate, and dynamometric assessment of the handgrip strength. Blood samples taken under fasting conditions were biochemically examined. Arterial hypertension was defined according to the guidelines of the American College of Cardiology/American Heart Association^20^. Diabetes mellitus was characterized by a fasting glucose concentration of ≥ 7.0 mmol/L or self-reported history of physician diagnosis of diabetes mellitus or history of drug treatment of diabetes. Depression was assessed applying the Center for Epidemiologic Studies Depression Scale Scoresheet^21^. We calculated the estimated glomerular filtration rate using the chronic kidney disease Epidemiology Collaboration equation^22^. We applied the Guidelines for Accurate and Transparent Health Estimates Reporting (GATHER statement guidelines)^23^. The UVOS design was similar to the design of the Ural Eye and Medical Study (UEMS) which has been described in detail previously^14, 24^.

The ophthalmological examinations, performed for all participating individuals, consisted of automated refractometry, measurement of presenting, uncorrected and best corrected visual acuity (using the results of automatic refractometry and refining it by subjective comparison), static perimetry (PTS 1000 Perimeter, Optopol Technology Co., Zawercie, Poland; screening test program with 82 test points and an extension of 50° in all directions), anterior segment imaging using the Scheimflug camera (Pentacam HR, Typ70900, OCULUS, Optikgeräte GmbH Co., Wetzlar, Germany) for measurements of the cornea, anterior chamber and lens, biometric measurement of the axial length by sonography (Ultra-compact A/B/P ultrasound system, Compact touch; Quantel Medical, Cournon d'Auvergne, France, US-4000, Nidek, Japan), slit lamp biomicroscopy of the anterior and posterior ocular segment, photography of the cornea and lens (Topcon slit lamp and camera, Topcon Corp., Tokyo, Japan), non-contact tonometry (Tonometer Kowa KT-800, Kowa Company Ltd., Hamamatsu City, Japan, Tonoref III, Nidek, Japan, PT 100 Recharging Base,Reichert, USA), examination for the presence of lens pseudoexfoliation after medical mydriasis, photography of the optic disc and macula (VISUCAM 500, Carl Zeiss Meditec AG, Jena, Germany, Optomed smartscope EY4, Finland), and spectral-domain optical coherence tomography (RS-3000 Edvance,, NIDEK co., Ltd., Aichi Japan, DRI OCT Triton (plus), Topcon, Japan) of the optic nerve head and macula.

Using a statistical software package (SPSS for Windows, version 25.0, SPSS, Chicago, IL), we determined the mean value of axial length and of its determinants (presented as mean ± standard error or as mean and 95% confidence intervals (CI)) and performed a univariate analysis of the relationships between axial length and other systemic and ocular parameters. It was followed by a multivariable linear regression analysis with axial length as the dependent parameter and as independent variables all those parameters that were significantly correlated with axial length in the univariate analyses. In a step-by-step procedure, we first dropped those independent variables which showed a high collinearity, as measured by the variance inflation factor. We then dropped in a step-by-step procedure those parameters out of the list of independent variables which were no longer significantly associated with axial length. We calculated the odds ratios (ORs) and their 95% CIs. All *P*-values were two-sided and considered statistically significant when the values were less than 0.05. Since the main study parameter was axial length and not refractive error, we did not exclude pseudophakic or aphakic individuals. Only one randomly selected eye per study participant was included into the statistical analysis.

## Results

**Figure 1:**
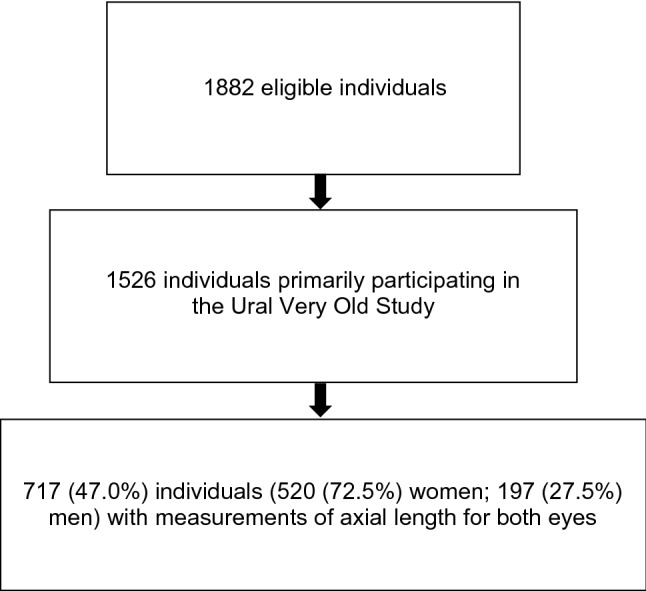
Flow chart of the population of the Ural Very Old Study.

Out of 1526 individuals primarily participating in the UVOS and undergoing the standardized interview at their homes, the present investigation included 717 (47.0%) individuals for whom measurements of axial length for both eyes had been performed in the hospital (Table 2; Fig. 1). The reasons for individuals not participating in the study were mainly their physical inability to be transported to the hospital and undergo the hourlong series of examinations in the hospital, or their lack of interest in participating in the study. The individuals with axial length assessment as compared with those without this examination were significantly younger (88.0 ± 2.6 years versus 88.6 ± 3.1 years; *P* > 0.001) and a significantly higher level of education (4.96 ± 1.95 versus 4.14 ± 2.03; *P* > 0.001), while they did not differ significantly in sex (*P* = 0.11).

**Table 2:** Demographic parameters of the study population.

Age	88.0 ± 2.6 years (median: 87 years; range: 85–98 years)
Ethnicity	295 (41.1%) individuals of Russian ethnicity, 300 (41.8%) Tartars, 72 (10.0%) Bashkirs, 6 (0.8%) Chuvash, 6 (0.8%) Mari, and 38 (5.3%) others 999999999999
Family status	101 (14.1%) were living in a joint family, 69 (9.6%) in a nuclear family, 306 (42.7%) were living alone, and 236 (32.9%) together with another family member
Level of education	Nine (1.3%) participates were illiterate, 98 (13.7%) had passed the 6th class, 144 (20.1%) the 8th class, 34 (4.7%) the 10th class and 30 (4.%) the 12th class; 177 (24.7%) had undergone a specialized secondary education, 217 (30.3%) were graduates and 3 (0.4%) postgraduates
Self-reported income	Below the poverty line: 147 (20.5%) participants; average income: 557 /77.7%) individuals; above average: 8 (1.1%) participants
Body height	156 ± 9 cm (range 113–181 cm)
Body weight	66.0 ± 11.7 kg (range 32–102 kg)
Body mass index	27.0 ± 4.4 kg/m^2^ (range 14.7–54.6 kg/m^2^)

**Figure 2:**
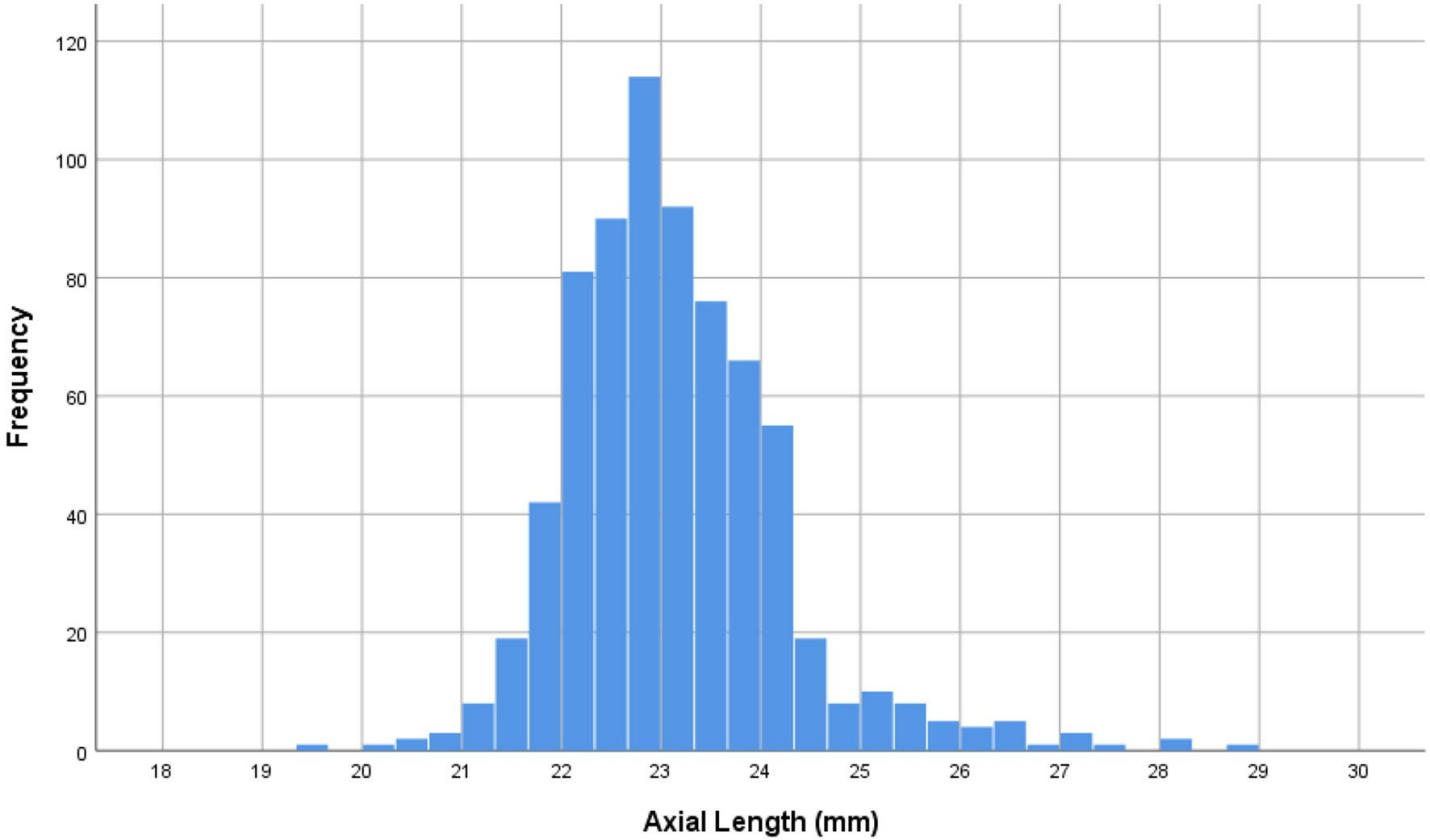
Histogram showing the distribution of axial length in the Ural Very Old Study.

**Figure 3:**
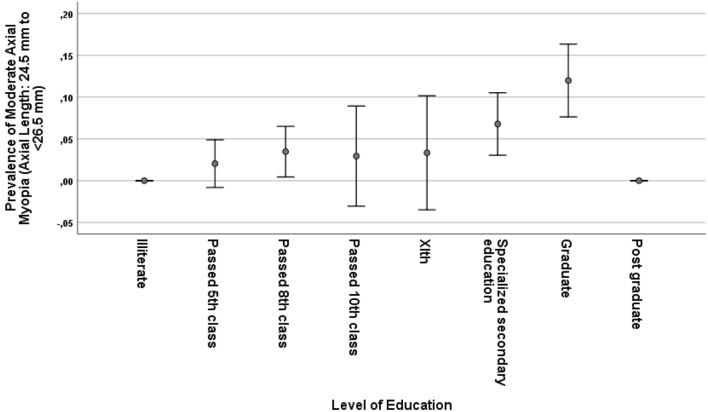
Graph showing the distribution of the prevalence of moderate myopia (axial length 24.5 mm to < 26.5 mm) in the Ural Very Old, stratified by the level of education.

Mean axial length was 23.1 ± 1.1 mm (median 22.97 mm; range 19.37–28.89 mm) and 23.1 ± 1.2 mm (median 23.00; range 19.50–28.84 mm) in the right eyes and left eyes, respectively (Fig. 2). Both eyes did not differ significantly in axial length (*P* = 0.05). Prevalence of moderate myopia (arbitrarily defined by an axial length of 24.5 to < 26.5 mm) and high myopia (arbitrarily defined by an axial length of ≥ 26.5 mm) were 47/717 (6.6%; 95% CI 4.7, 8.4) and 10/717 (1.4%; 95% CI 0.5, 2.3), respectively. In univariate analysis, a longer axial length was associated (*P* < 0.05) with the systemic parameters of female sex, rural region of habitation, taller body height, higher body weight and lower body mass index, higher level of education (Fig. 3), more frequent history of asthma, less frequent history of dementia, lower serum concentration of high-density lipoproteins and cholesterol, higher dynamometric hand grip force, higher mini-mental test score, and with the ocular parameters of more myopic refractive error (spherical and spherical equivalent), higher cylindrical refractive error, lower corneal refractive power, and higher prevalence of dry eye syndrome (defined by a dry eye score ≥ 8 and Schirmer’s test ≤ 5 mm) (Table 1).

In the multivariable analysis, we dropped due to collinearity the parameters of body weight (variance inflation factor (VIF): 123), and we dropped due to a lack of statistical significance the parameters of serum concentrations of cholesterol (*P* = 0.88) and high-density lipoproteins (*P* = 0.62), dynamometric hand grip force (*P* = 0.98), body mass index (*P* = 0.81), sex (*P* = 0.81), history of dementia (*P* = 0.22), mini mental test score (*P* = 0.24), region of habitation (*P* = 0.19), and diagnosis of dry eye disease (*P* = 0.06). In the final model, longer axial length was associated (correlation coefficient r 0.50) with taller body height (*P* < 0.001), higher level of education (*P* = 0.002), lower corneal refractive power (*P* < 0.001), in addition to a more myopic spherical refractive error (*P* < 0.001) (Table 3). The associations remained statistically significant after correcting the *P*-values by Bonferroni´s method to adjust for performing multiple statistical comparisons. In that model, taller body height (per centimeter) was associated with an increase in axial length by 0.02 mm, higher level of education (per each level out of 8 levels) was associated with an increase in axial length by 0.07 mm, higher corneal refractive power (per diopter) was associated with a decrease in axial length by 0.23 mm, and more myopic refractive error was associated with an increase in axial length by 0.09 mm (Table 3). Axial length was not significantly associated in that model with age (*P* = 0.27), sex (*P* = 0.55), region of habitation (*P* = 0.29), BCVA (*P* = 0.62), and intraocular pressure (*P* = 0.12). If the parameter of body height was dropped, longer axial length was associated with male sex (beta 0.12; B 0.28; 95% CI 0.10, 0.45; *P* = 0.002).

**Table 3:** Associations (multivariable analysis; correlation coefficient r: 0.50) between axial length and other parameters in the Ural Very Old Study.

Parameter	Non-standardized regression coefficient B	95% confidence interval	Standardized regression coefficient B	*P*-value
Body height (cm)	0.02	0.01, 0.03	0.16	< 0.001
Level of education	0.07	0.02, 0.11	0.12	0.002
Corneal refractive power (diopters)	− 0.23	− 0.28, − 0.18	0.35	< 0.001
Spherical refractive error (diopters)	− 0.09	− 0.12, − 0.07	− 0.25	< 0.001

The prevalence of moderate myopia was significantly (univariate analysis) associated with body height (*P* = 0.02), level of education (*P* < 0.001), corneal refractive power (*P* = 0.01), and higher myopic refractive error (*P* = 0.004), but not with sex (*P* = 0.17), region of habitation (*P* = 0.99) or BCVA (*P* = 0.10). In multivariable binary analysis, a higher prevalence of moderate myopia remained to be significantly associated with a higher educational level (OR 1.39; 95% CI 1.09, 1.68; *P* = 0.007), lower corneal refractive power (OR 0.77; 95% CI 0.63, 0.94; *P* = 0.01) and more myopic refractive error (OR 0.82; 95% CI 0.74, 0.91; *P* < 0.001), while it was no longer associated with body height (*P* = 0.36).

The prevalence of high axial myopia was not significantly (univariate analysis) associated with body height (*P* = 0.18), level of education (*P* = 0.10), sex (*P* = 0.60), region of habitation (*P* = 0.99), corneal refractive power (*P* = 0.56). It was significantly correlated only with higher myopic refractive error (OR 0.82; 95% CI 0.72, 0.94; *P* = 0.002).

## Discussion

In our study on an old population recruited in a population-based manner, mean axial length was 23.1 ± 1.1 mm, with a mean prevalence of moderate myopia and high myopia of 6.6% and 1.4%, respectively. Longer axial length was associated with taller body height, higher level of education, lower corneal refractive power and more myopic refractive error. Higher prevalence of moderate myopia was correlated with higher level of education, lower corneal refractive power and more myopic refractive error, while the prevalence of high axial myopia was not correlated with any of these parameters, except for higher myopic refractive error.

These results cannot directly be compared with observations made in previous investigations, since a population with an age inclusion criterion of 85 + years as in our study has not been examined previously in such a context. In younger study populations, partially similar results were obtained as in our study. To cite an example, in the UEMS, performed on a study population aged 40 + years (mean:58.8 ± 10.6 years) and conducted in the same region as the present study, the mean axial length was 23.3 ± 1.1 mm, a value similar to the result in our study population^14^. The mean axial length as found in our study was larger than in some other population-based studies, such as in the Central India and Medical Study (CIEMS) (22.6 ± 0.91 mm) and in a rural South Indian population study (22.8 ± 0.8 mm), they were similar to the values reported from a Mongolian population (23.1 ± 1.2 mm) and the Singaporean Tanjong Pagar Study (23.2 ± 1.2 mm), and they were lower than the mean axial length found in the Los Angeles Latino Eye Study (23.4 ± 1.1 mm), the Beijing Eye Study (23.3 ± 1.1 mm), the Beaver Dam Eye Study (23.7 ± 1.2 mm) and the EPIC-Norfolk study from England (23.8 ± 1.2 mm^8, 10–13, 25, 26^.

The prevalence of moderate myopia (axial length 24.5 to < 26.5 mm) was significantly lower in our study sample than in the younger population of the UEMS [6.6% (95% CI 4.7, 8.4) versus 9.7% (95% CI 9.0, 10.5)], and it was also lower than in other studies such as the Beijing Eye Study with a prevalence of moderate myopia (with the same definition) of 8.6% (95% CI 7.3, 9.2)^13^. It was higher than in the CIEMS (1.5%; 95% CI 1.2, 1.7).

The prevalence of high myopia (axial length > 26.5 mm) was similar in the UEMS and in the present study [1.4% (95% CI 1.1, 1.7) versus 1.4% (95% CI 0.5, 2.3), while it was in both studies lower than in the Beijing Eye Study (minimal age of 40 years) with a prevalence of 1.7% (95% CI 1.3, 2.2)^13^. It was markedly higher than in the population of the CIEMS (0.4%; 95% CI 0.3, 0.6). A reason for the discrepancy between the study populations in the prevalence in high myopia may be differences in the prevalence of overall myopia, since the CIEMS as compared to the present study had a lower prevalence also of moderate myopia. Another reason may be a survival factor, with a potentially lower life expectancy for highly myopic individuals in the very rural region in Central India. The cause for the differences in the prevalence of moderate myopia may be the association between longer axial length and higher level of education, with the lowest mean level of education in the very rural study region of the CIEMS. It would fit with the observation of a marked increase in the prevalence of axial myopia in the young generation in China, parallel to a profound increase in the educational activities of school children in China^27^.

The association between longer axial length and higher educational level is a universal phenomenon, since it was reported, besides from our investigation, also from studies performed in other countries^12, 13, 28, 29^. In our study, the prevalence of high myopia, in contrast to the prevalence of moderate myopia, was not significantly related to the level of education. As a corollary, the prevalence of high myopia in the Beijing Eye Study on adults aged 40 + years was associated with a lower level of education^13^. It fits with the results of a recent meta-analysis, in which the prevalence of high myopia in adults was not, however, the prevalence of high myopia in school children was, associated with education-related parameters^30^.

Longer axial length was associated with taller body height in our study as well as in the UEMS, and as also reported from the Singaporean Tanjong Pagar Study, the Icelandic Reykjavik Eye Study, the Burmese Meiktila Eye Study and the Beijing Eye Study^8, 13, 31, 32^. It fits with the observation that the prevalence of primary angle-closure glaucoma was associated with shorter body height in previous studies^33^.

In contrast to previous studies on younger populations, such as the Beijing Eye Study, the CIEMS, a study from Mongolia and the UEMS, axial length did not increase with older age in our study population^10, 12–14^. In other studies, however, axial length decreased with older age, or it was not related at all^8, 10, 11^. Reasons for the discrepancies may be when the marked increase in axial myopia in association with more time spent indoors versus outdoors and the more profound educational activities started in the various countries.

Axial length was not related with IOP in our study, in contrast to the UEMS and the Japanese Kumejima study, the Japanese Tajimi Study and the Chinese Handan Study^14, 34–36^. As in our study, axial length was unrelated to IOP in the Los Angeles Latino Eye Study^37^. While the causes for a potential association between axial length and IOP have remained elusive, it has been discussed whether higher IOP leads to longer axial length or whether axial elongation-associated factors lead to an increase in IOP. Axial length was neither associated with BCVA in our study, while in the UEMS, BCVA showed a curvilinear relationship with axial length, with an improvement of BCVA from short axial length to medium axial length values, and deterioration of BCVA towards long axial length readings^14^. The marked difference in age between both study population may potentially be the reason for the discrepancy between both studies since axial myopia is partially protective against age-related macular degeneration and diabetic retinopathy^3, 4^.

When the findings obtained in the present investigation are discussed, its limitations should be considered. First, although 1526 out of 1882 eligible individuals participated in the Ural Very Old Study, resulting in a participation rate of 81%, only 717 (47.0%) individuals underwent the clinical examination including biometric axial length measurements. The individuals with biometry, as compared to those without biometry, were significantly younger and had a significantly higher educational level. These differences might have led to a bias. If one considers, however, the relatively old age of our study population with a minimum age of 85 years and the multimorbidity often occurring in that age, the participation rate for the clinical examinations in our study population may be acceptable. Second, the study regions of the UVOS were characteristic for Southern Russia in terms of demography, geography and climate. In terms of the ethnic background, the percentage of Russians was lower in our study population than in populations from North-Western Russia and Central Russia. In the multivariable analysis, however, the ethnic background was not associated with axial length, so that the relatively high percentage of non-Russians on the total study population might not have markedly influenced the results. Strengths of our investigation are that it is the first population-based investigation on axial length in a very old population, that also the inhabitants of all retirement homes in the study regions were included into the study, and that a multitude of systemic parameters was assessed and included in the statistical multivariable analysis. Since such a study population had not been examined previously, all findings were principally novel, while of special interest was the finding that the prevalence of moderate axial myopia was lower than, and the prevalence of high axial myopia was similar as, in a younger population in the same study regions.

In conclusion, in this old, multi-ethnic study population from rural and urban Russia, the prevalence of moderate axial myopia (6.6% versus 9.7%) was lower than, and the prevalence of high axial myopia (1.4% versus 1.4%) was similar as, in a corresponding study on a younger population from the same Russian region. The prevalence rates were higher than in rural Central India (1.5% and 0.4%, respectively). As in other, younger, populations, axial length and moderate myopia prevalence increased with higher educational level, while high myopia prevalence was independent of the educational level. The association between longer axial length and taller body height agrees with the notion of larger eyes in taller individuals.
